# *Lacisediminihabitans profunda* gen. nov., sp. nov., a member of the family *Microbacteriaceae* isolated from freshwater sediment

**DOI:** 10.1007/s10482-019-01347-8

**Published:** 2019-10-18

**Authors:** Ye Zhuo, Chun-Zhi Jin, Feng-Jie Jin, Taihua Li, Dong Hyo Kang, Hee-Mock Oh, Hyung-Gwan Lee, Long Jin

**Affiliations:** 1 College of Biology and the Environment, Co-Innovation Centre for Sustainable Forestry in Southern China, Nanjing Forestry University, Nanjing, 210037 China; 2 Department of Bio-Molecular Science, KRIBB School of Bioscience, Korea University of Science and Technology (UST), 217 Gajeong-ro, Yuseong-gu, Daejeon, Republic of Korea; 3 Industrial Biomaterial Research Center, Korea Research Institute of Bioscience and Biotechnology (KRIBB), Daejeon, 34141 Republic of Korea; 4 Cell Factory Research Centre, Korea Research Institute of Bioscience and Biotechnology (KRIBB), Daejeon, 34141 Republic of Korea

**Keywords:** *Lacisediminihabitans*, *Lacisediminihabitans profunda*, CHu50b-6-2, Sediment

## Abstract

A novel Gram-stain-positive bacterial strain, CHu50b-6-2^T^, was isolated from a 67-cm-long sediment core collected from the Daechung Reservoir at a water depth of 17 m, Daejeon, Republic of Korea. The cells of strain CHu50b-6-2^T^ were aerobic non-motile and formed yellow colonies on R2A agar. The phylogenetic analysis based on 16S rRNA gene sequencing indicated that the strain formed a separate lineage within the family *Microbacteriaceae*, exhibiting 98.0%, 97.7% and 97.6% 16S rRNA gene sequence similarities to *Glaciihabitans tibetensis* KCTC 29148^T^, *Frigoribacterium faeni* KACC 20509^T^ and *Lysinibacter cavernae* DSM 27960^T^, respectively. The phylogenetic trees revealed that strain CHu50b-6-2^T^ did not show a clear affiliation to any genus within the family *Microbacteriaceae*. The chemotaxonomic results showed B1*α* type peptidoglacan containg 2, 4-diaminobutyric acid (DAB) as the diagnostic diamino acid, MK-10 as the predominant respiratory menaquinone, diphosphatidylglycerol, phosphatidylglycerol, and an unidentified glycolipid as the major polar lipids, anteiso-C_15:0_, iso-C_16:0_, and anteiso-C_17:0_ as the major fatty acids, and a DNA G + C content of 67.3 mol%. The combined genotypic and phenotypic data showed that strain CHu50b-6-2^T^ could be distinguished from all genera within the family *Microbacteriaceae* and represents a novel genus, *Lacisediminihabitans* gen. nov., with the name *Lacisediminihabitans profunda* sp. nov., in the family *Microbacteriaceae*. The type strain is CHu50b-6-2^T^ (= KCTC 49081^T^ = JCM 32673^T^).

**Electronic supplementary material:**

The online version of this article (10.1007/s10482-019-01347-8) contains supplementary material, which is available to authorized users.

## Introduction

Since Park et al. (1993) proposed the family *Microbacteriaceae*, 56 genera have been described validly in this family at the time of writing (http://www.bacterio.net/; Parte 2018). Members of the family *Microbacteriaceae* are widely distributed in nature including soil, freshwater, groundwater, cyanobacterial mats, the rhizosphere and phyllosphere of plants, air and ice samples, ponds in Antarctica, sludge, seawater, sediment, seaweed, and seafood (Dias and Bhat 1962; Männistö et al. 2000; Reddy et al. 2003; Lee 2007; Kim et al. 2008; Kim and Lee 2011; Shin et al. 2011; Jang et al. 2012; Park et al. 2012; Schumann et al. 2012; Jin et al. 2013; Lai et al. 2015). During an investigation on iron and sulfur oxidizing microbial diversity in the sediment of a eutrophic freshwater reservoir (Jin et al. 2017), a strain designated CHu50b-6-2^T^ was isolated from the freshwater sediment of the Daechung Reservoir. Herein, we describe the phylogenetic, genetic, phenotypic and chemotaxonomic characteristics of this novel strain, which is proposed to represent a new genus within the family *Microbacteriaceae* by using a polyphasic approach.

## Materials and methods

### Isolation, morphological and physiological characterization

Strain CHu50b-6-2^T^ was recovered from a 67-cm-long sediment core (36° 22′ 30″ N, 127° 33′ 58″ E) collected from the Daechung Reservoir at a water depth of 17 m in Daejeon, South Korea. 1 g sediment sample was applied to serial dilution method. A 100 μl sub-sample (10^−6^ or 10^−7^) of the suspended material was spread onto modified 1/10 R2A agar (L^−1^: 0.05 g peptone, 0.05 g yeast extract, 0.05 g casamino acid, 0.05 g dextrose, 0.05 g soluble starch, 0.03 g K_2_HPO_4_, 0.005 g MgSO_4_, 0.03 g sodium pyruvate, and 15 g agar) and incubated at room temperature (25 °C) for 4 weeks. One yellow colony, designated as CHu50b-6-2^T^, was isolated and subcultivated on R2A agar at 30 °C for further analysis. The colony characteristics were determined after growing for 5 days at 30 °C on R2A agar. Gram staining was performed using a Gram stain kit (Becton–Dickinson) and 3% KOH solution. The cell morphology and motility were examined under a phase-contrast microscope (Nikon Eclipse 80i microscope, 1000 × magnification) and a transmission electron microscope (CM20, Philips; Netherlands) after negative staining with 2% (w/v) uranyl acetate using cells grown for 48 h on R2A agar.

The cell growth was checked on R2A agar, trypticase soy agar (TSA; Difco), Luria–Bertani (LB; Difco) medium, and nutrient agar. The growth temperature range was checked by incubating at 4, 8, 15, 20, 30, 37, and 45 °C on R2A agar. Salt tolerance was performed by adding different concentrations of NaCl to R2A agar. The pH growth was determined in R2A broth with a pH range of 5–11 at intervals of 1 pH unit. Different biological buffers were applied to adjust the pH values: Na_2_HPO_4_/NaH_2_PO_4_ buffer for pH 5–7 and Na_2_CO_3_/NaHCO_3_ buffer for pH 8–11 (Bates and Bower 1956; Gomori 1955). The oxidase activity was checked using 1% tetramethyl-*p*-phenylenediamine (Tarrand and Groschel 1982), and the catalase activity was checked using 3% H_2_O_2_. We used the API 20NE, ID 32 DN, API ZYM kits (bioMérieux), and Biolog GN2 MicroPlate to determine carbon source utilization and to do enzyme activity assays as well as additional physiological tests following the manufacturer’s instructions. Duplicate antibiotic-susceptibility tests were conducted using filter-paper discs (6 mm) containing the following: amikacin (30 µg ml^−1^), ampicillin/sulbactam (20 µg ml^−1^, 1:1), chloramphenicol (30 µg ml^−1^), erythromycin (30 µg ml^−1^), gentamicin (30 µg ml^−1^), kanamycin (30 µg ml^−1^), lincomycin (15 µg ml^−1^), nalidixic acid (30 µg ml^−1^), rifampicin (30 µg ml^−1^), spectinomycin (25 µg ml^−1^), streptomycin (25 µg ml^−1^), teicoplanin (30 µg ml^−1^), tetracycline (30 µg ml^−1^), and vancomycin (30 µg ml^−1^). The discs were placed on R2A plates spread with a culture of strain CHu50b-6-2^T^ and were then incubated at 30 °C for 2 days. Susceptibility was recorded as positive at zones with diameters greater than 10 mm.

### Chemotaxonomic characterisation

For fatty acid profiling, strain CHu50b-6-2^T^ was cultured on R2A agar for 48 h to the late exponential phase. Harvesting of the cell mass was standardized in the instruction of MIDI (http://www.microbialid.com/PDF/TechNote_101.pdf). Separation and identification of the fatty acids were performed by GC (Hewlett Packard 6890), and the TSBA 6 database provided the Sherlock software 6.1. Extraction of isoprenoid quinone was carried out following the method described by Komagata and Suzuki (1987), and the analysis was done by HPLC (Shimadzu) with an YMC-Pack ODS-A column. Extraction and identification of polar lipids were done using two-dimensional TLC following the method described by Tindall (1990). The isomer of diaminopimelic acid (DAP) in the cell wall was analyzed using the method described by Hasegawa et al. (1983). The cell-wall peptidoglycan was extracted and identified using TLC after hydrolysis with 6 M HCl at 100 °C for 18 h (Komagata and Suzuki 1987). Genomic DNA was extracted using a commercial genomic DNA-extraction kit (FastDNA™ SPIN kit). The purity of the extracted DNA was then examined on a ND2000 spectrometer (Nanodrop Technologies, Inc.). DNA G + C contents (mol%) were analyzed by HPLC after hydrolysis as described by Tamaoka and Komagata (1984). Three reference strains were used: *Glaciihabitans tibetensis* KCTC 29148^T^ was obtained from the KCTC (Korean Collection for Type Cultures), *Frigoribacterium faeni* KACC 20509^T^ from the KACC (Korean Agricultural Culture Collection), and *Lysinibacter cavernae* DSM 27960^T^ from the DSMZ (German Collection of Microorganisms and Cell Cultures).

### Molecular characterization

The 16S rRNA gene was amplified by PCR as described previously (Ren et al. 2018) using the universal bacterial primer sets, 27F (5′-AGA GTT TGA TCM TGG CTC AG-3′; *Escherichia coli* position 8–27) and 1492R (5′-TAC GGY TAC CTT GTT ACG ACT T-3′; *E. coli* position 1492–1510, were used (Lane 1991). The purified PCR products then were sequenced with the BigDye Terminator v3.1 Cycle Sequencing kit (Applied Biosystems). Whole genome was sequenced via the Illumina HiSeq platform. The genome was assembled by the CLC assembler (CLC-Assembly-Cell-5.1.1), and the gene annotation was performed by the PATRIC 3.5.36 (hps://www.patricbrc.org). The average nucleotide identity (ANI) was calculated using OrthoANI tool in the EZBioCloud (Lee et al. 2016). To get the full 16S rRNA gene, the sequencing primers 27F, 785F (5′-GGA TTA GAT ACC CTG GTA-3′), 800R (5′-TAC CAG GGT ATC TAA TCC-3′), and 1492R for the sequence analysis, were used (Lane 1991). The phylogenetic neighbors of strain CHu50b-6-2^T^ were identified, and the pairwise similarities of the 16S rRNA gene sequences were calculated with EzBioCloud (Yoon et al. 2017). The retrieved 16S rRNA gene sequences were aligned using the clustal x program (Thompson et al. 1997). Evolutionary distances were calculated based on Kimura’s two-parameter model (Kimura 1980). Phylogenetic trees were reconstructed with mega version 7.0 (Kumar et al. 2016) applying the neighbor-joining (Saitou and Nei 1987), maximum-likelihood (Felsenstein 1981) and maximum parsimony (Fitch 1971) algorithms. The bootstrap values were based on 1000 replicates (Felsenstein 1985). The housekeeping gene, *rec*A gene encoding DNA recombinase A, was applied do delineate our strain more clearly from its close species. Housekeeping genes are useful for species identification as phylogenetic markers. The primer sets, *rec*A-F (5′-GTT CTC YTT RCC CTG NCC-3′) and *rec*A-R (5′-GAR TCS TCS GGW AAG ACB AC-3′), were used for amplifying and sequencing (Katayama et al. 2009). The PCR amplification conditions were as following: 95 °C for 5 min, 30 cycles of 95 °C for 1.5 min, 55 °C for 1 min and 72 °C for 1 min and final extension for 10 min at 72 °C. To determine genomic relatedness, DNA-DNA hybridisation experiment was carried out between strain CHu50b-6-2^T^ and type strains of *G. tibetensis*, *F. faeni* and *L. cavernae*, which showed over 97% of 16S rRNA gene similarities to novel strain. The hybridisation test was carried out as described by Ezaki et al. (1989), and salmon sperm DNA (Sigma; D7656) was used as a control.

## Results and discussion

Strain CHu50b-6-2^T^ formed yellow colonies within 48 h on R2A agar at 30 °C. While cell growth occurred at temperatures ranging from 4 to 30 °C, no growth was observed at 37 °C. Growth was observed at pH 6 to 10, but no growth was observed at pH 5 or 11. The colonies were convex and circular with entire edges. The cells were found to be Gram-stain-positive, oxidase-negative, catalase-positive, non-motile, and rod shaped (Supplementary Fig. 1). The cells were observed to assimilate *N*-acetyl-d-galactosamine, *N*-acetyl-glucosamine, l-arabinose, 2,3-butanediol, d-cellobiose, dextrin, d-fructose, d-galactose, d-gluconic acid, d-glucose, glycerol, inosine, inositol, *α*-keto butyric acid, dl-lactate, lactulose, maltose, mannitol, d-melibiose, pyruvic acid methyl ester, d-raffinose, rhamnose, d-ribose, d-sorbitol, sucrose, turanose, thymidine, uridine, and xylitol but not the rest (API 20NE, API ID 32GN test strips and Biolog GN2 MicroPlate). The cells were found to be positive for the following enzyme activities (API ZYM test strip): acid phosphatase, cystine arylamidase, esterase (C4), esterase lipase (C8), *α*-galactosidase, *β*-galactosidase, *α*-glucosidase, *β*-glucosidase, leucine arylamidase, naphtol-AS-BI-phosphohydrolase, and valine arylamidase but the cells were found to be negative for the following enzyme activities: *N*-acetyl-*β*-glucosaminidase, alkaline phosphatase, *α*-chymotrypsin, *α*-fucosidase, *β*-glucuronidase, lipase (C14), *α*-mannosidase and trypsin (Table 1). The cells were found to be susceptible to amikacin (30 µg ml^−1^), chloramphenicol (30 µg ml^−1^), erythromycin (30 µg ml^−1^), kanamycin (30 µg ml^−1^), lincomycin (15 µg ml^−1^), rifampicin (30 µg ml^−1^), spectinomycin (25 µg ml^−1^), streptomycin (25 µg ml^−1^), teicoplanin (30 µg ml^−1^), and vancomycin (30 µg ml^−1^) but resistant to ampicillin/sulbactam (1:1; 20 µg ml^−1^), nalidixic acid (30 µg ml^−1^), gentamicin (30 µg ml^−1^), and tetracycline (30 µg ml^−1^). Strain CHu50b-6-2^T^ could be differentiated from the closest species *G. tibetensis* by assimilating *N*-acetyl-glucosamine, l-arabinose, d-glucose, maltose, and d-mannitol and by activities of nitrate reduction, arginine dihydrolase, oxidase, *α*-chymotrypsin, lipase (C14), and trypsin; from *F. faeni* by assimilating *N*-acetyl-glucosamine and not assimilating citrate, by activities of arginine dihydrolase, alkaline phosphatase, and motility; from *L. cavernae* by not assimilating gluconate and d-mannose, by activities of nitrate reduction, arginine dihydrolase, oxidase, cystine arylamidase, *α*-galactosidase, *β*-galactosidase, and lipase (C14).

**Table 1:** Phenotypic and chemotaxonomic characteristics distinguishing strain CHu50b-6-2^T^ from three closest members

Characteristics	1	2	3	4
Isolation source	Sediment	Glacier water	Hay dust	Soil
Morphology	Rod	Short rod	Irregular rod	Rod
Colony colour	Yellow	Pale yellow	Pale yellow	Brilliant yellow
Motility	–	–	+	–
Nitrate reduction	+	–	–	–
Oxidase/catalase	−/+	+/+	−/+	+/+
Arginine dihydrolase	–	+	+	+
NaCl tolerance range (w/v, %)	0–2	0–4.5^a^	0–7^b^	0–5^c^
Carbon utilization:				
*N*-Acetyl-glucosamine	w	–	–	+
l-Arabinose	+	–	+	+
Citrate	–	–	+	–
Gluconate	–	–	–	+
d-Glucose	+	–	+	+
Maltose	+	–	+	+
d-Mannitol	+	–	+	+
d-Mannose	–	–	–	+
Enzyme activity:				
Alkaline phosphatase	–	–	+	–
*α*-Chymotrypsin	–	w	+	–
Cystine arylamidase	+	+	+	–
*α*-Galactosidase	+	+	+	–
*β*-Galactosidase	+	+	+	–
Lipase (C14)	–	w	–	w
Trypsin	–	w	–	–
Respiratory quinones	MK-10	MK-11, 10, 9^a^	MK-9^b^	MK-11, 10, 9^c^
Polar lipids	DPG, PG, GL, L1, L2, L3	DPG, PG, GL, L1, L2, L3, L4^c^	DPG, PG, GL, L1^c^	DPG, PG, GL, L1, L2, PL1, PL2, PL3, PL4^c^
DNA G + C content (mol%)	67.3	64.1^a^	71^b^	62.6^c^

Strains: 1, CHu50b-6-2^T^; 2, *G. tibetensis* KCTC 29148^T^; 3, *F. faeni* KACC 20509^T^; 4, *L. cavernae* DSM 27960^T^. All data were from this study, unless indicated. All strains were observed to be positive for activities of acid phosphatase, esterase (C4), esterase lipase (C8), *α*-glucosidase, *β*-glucosidase, leucine arylamidase, naphthol-AS-BI-phosphohydrolase, and valine arylamidase; All strains were observed to be negative for activities of indole production, glucose acidification, urease, gelatin hydrolysis, *N*-acetyl-*β*-glucosaminidase, *α*-fucosidase, *β*-glucuronidase, and α-mannosidase; carbon assimilation of adipate, caprate, malate, and phenyl acetate. DPG, diphosphatidylglycerol; PG, phosphatidylglycerol; GL, unidentified glycolipid; PL, unidentified phospholipid; L, unidentified lipid. −, negative; +, positive; w, weakly positive

^a^Data taken from Li et al. (2014)

^b^Kämpfer et al. (2000)

^c^Tuo et al. (2015)

The draft genome sequence of strain CHu50b-6-2^T^ was deposited at DDBJ/EMBL/GenBank with the accession number PRJNA559971. The draft genome of strain CHu50b-6-2^T^ was of 4,022,930 bp, containing 175 contigs, of which the largest was of 845,903 bp. The genome encoded 3975 genes, including 48 tRNAs and 7 rRNAs. The N50 value was 413,391 and the sequencing depth of coverage was 570X. The DNA G + C content calculated from the draft genome sequence was 67.3 mol% (Table S1). The ANI values of strain CHu50b-6-2^T^ with *G. tibetensis* MP203^T^ and *F. faeni* 801^T^ were 73.1 and 73.4, respectively.

**Fig. 1:**
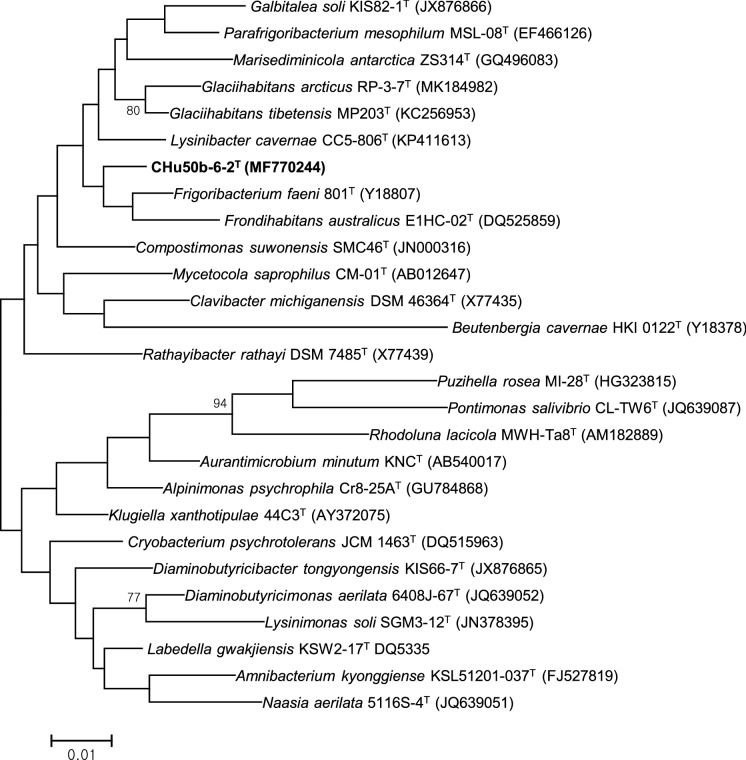
Phylogenetic tree based on 16S rRNA gene sequences using maximum-likelihood method showing position of strain CHu50b-6-2^T^ among type species within the family *Microbacteriaceae.* Numbers at branching points refer to bootstrap values (1000 resamplings, only values above 70% shown). *Beutenbergia cavernae* HKI 0122^T^ (Y18378) was used as an outgroup. Bar, 1 substitutions per 100 nt

**Fig. 2:**
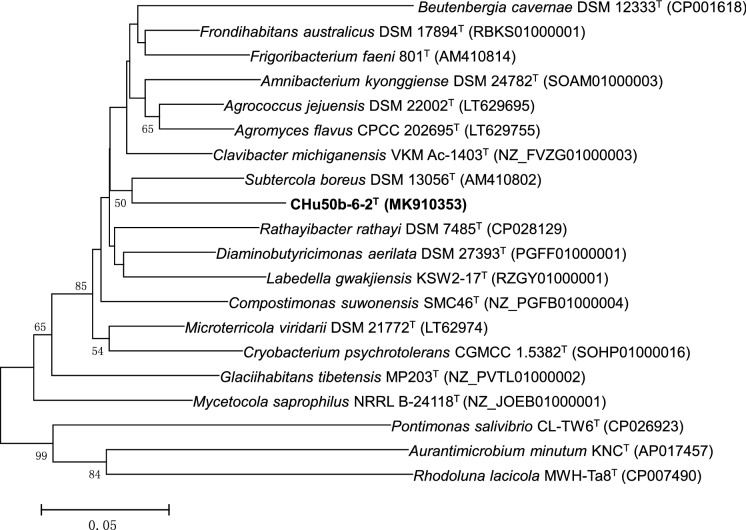
Phylogenetic tree of *rec*A gene using maximum-likelihood method showing positions of strain CHu50b-6-2^T^ and related taxa. *Beutenbergia cavernae* DSM 12333^T^ (CP001618) was used as an outgroup. Bar, 5 substitutions per 100 nt. Only bootstrap values above 50% are shown (1000 resamplings) at branching points

**Fig. 3:**
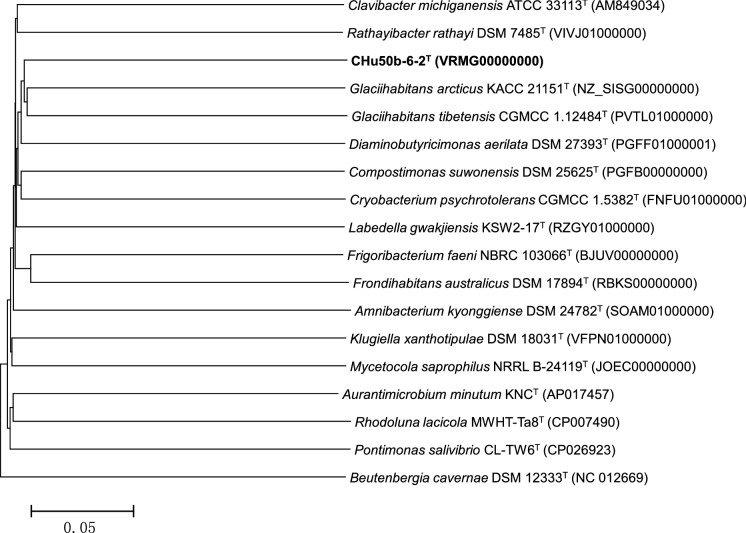
upgma dendrogram based on ANI values of genomic sequences showing the positions of strain CHu50b-6-2^T^ among the type species within the family *Microbacteriaceae*. *Beutenbergia cavernae* DSM 12333^T^ was used as an outgroup. *Beutenbergia cavernae* DSM 12333^T^ (NC 012669) was used as an outgroup. Bar, 5% difference in ANI value

The almost-complete 16S rRNA gene sequence (approximately 1461 nt) of strain CHu50b-6-2^T^ was compared with those of representative species within the family *Microbacteriaceae*. Strain CHu50b-6-2^T^ showed over 97% 16S rRNA gene sequence similarities with *G. tibetensis*, *F. faeni*, *L. cavernae*, *F. endophyticum*, *Parafrigoribacterium mesophilum*, *Cryobacterium arcticum*, *F. salinisoli*, and *Homoserinimonas aerilata*, and less than 97% with the remaining members of the family *Microbacteriaceae*. According to maximum-likelihood, neighbour-joining, and maximum-parsimony tree analysis (Fig. 1 & Supplementary Fig. S2), the isolate was phylogenetically distinct from closely related members of the family *Microbacteriaceae*, especially with *G. tibetensis*, *F. faeni*, *L. cavernae* with which it showed 98.0%, 97.7%, and 97.6% 16S rRNA gene sequence similarities, respectively. And the genomic delineation between strain CHu50b-6-2^T^ and the type strains of *G. tibetensis*, *F. faeni*, and *L. cavernae* was supported by the DNA-DNA relatedness (the mean of triplicate experiments) data, for which our novel isolate showed DNA-DNA relatedness values of 31.2% (reciprocal 29.2%), 29.7% (reciprocal 33.1%), and 22.5% (reciprocal 24.9%) with *G. tibetensis* KCTC 29148^T^, *F. faeni* KACC 20509^T^ and *L. cavernae* DSM 27960^T^, respectively. For clearer delineation, the housekeeping gene, *rec*A gene was applied as phylogenetic marker. The *rec*A gene sequence of CHu50b-6-2^T^ had 88.8%, 88.2%, 86.2%, 85.9%, and 85.4% similarities with *Clavibacter michiganensis* VKM Ac-1403^T^, *Subtercola boreus* DSM 13056^T^, *Rathayibacter rathayi* DSM 7485^T^, *F. faeni* KACC 20509^T^, and *G. tibetensis* KCTC 29148^T^ respectively. Overall, phylogenetic analyses based on 16S rRNA, *rec*A genes and upgma dendrogram based on the ANI values of genomic sequences revealed groups that are in good agreement with the currently recognized genera (Figs. 1, 2, 3). The lower DNA-DNA hybridization values together with phylogenetic analysis revealed that strain CHu50b-6-2^T^ could not be clearly assigned to any species of the genus *Glaciihabitans*, *Frigoribacterium*, and *Lysinibacter*.

The G + C content of the genomic DNA was 67.3 mol%, and the major fatty acids were *anteiso*-C_15:0_ (46.6%), *iso*-C_16:0_ (29.7%), and *anteiso*-C_17:0_ (14.5%) (Table 2). The major predominant respiratory menaquinone was MK-10. The polar lipids consisted of diphosphatidylglycerol (DPG), phosphatidylglycerol (PG), an unidentified glycolipid (GL), and three unidentified lipids (L1, L2, and L3) (Supplementary Fig. 3). It is noteworthy that strain CHu50b-6-2^T^ had a large amount of *anteiso*-C_17:0_ (14.5%) compared to the closet members *G. tibetensis* KCTC 29148^T^ (3.6%), *F. faeni* KACC 20509^T^ (5.1%) and *L. cavernae* DSM 27960^T^ (1.8%), and a smaller amount of *iso*-C_14:0_ (1.6%) compared to *L. cavernae* DSM 27960^T^ (22.2%). Although the overall polar lipid patterns were very similar, there were some differences in the unidentified phospholipids and the unidentified lipids between CHu50b-6-2^T^ and the species *G. tibetensis*, *F. faeni* and *L. cavernae*. Strain CHu50b-6-2^T^ had B1*α* type pepdidoglycan structure, which differed from phylogenetically related genera *Glaciihabitans*, *Frigoribacterium*, and *Lysinibacter*. Together with some other physiological results, it could be concluded that the strain CHu50b-6-2^T^ differs from the close species *G. tibetensis*, *F. faeni* and *L. cavernae* (Tables 1, 2). And also, strain CHu50b-6-2^T^ can be differentiated from closely related genera within the family *Microbacteriaceae* on the basis of its chemotaxonomic characteristics such as fatty acids, polar lipids, menaquinones, and G + C content (Table 3). Therefore, it should be considered that the strain is not accommodated in any of known genera within the family *Microbacteriaceae*.

**Table 2:** Cellular fatty acid compositions (%) of strain CHu50b-6-2^T^ and related type strains

Fatty acids	1	2	3	4
Iso-C_14:0_	1.6	5.9	3.4	22.2
Iso-C_13:0_ 3 OH	tr	tr	2.6	tr
Anteiso-C_15:1_ A	2.7	1.5	tr	tr
Iso-C_15:0_	3.1	1.7	3.8	1.0
Anteiso-C_15:0_	46.6	57.9	38.0	27.2
Iso-C_14:0_ 3 OH	tr	tr	1.1	tr
C_14:0_ 2 OH	tr	4.2	17.0	tr
Iso-C_16:0_	29.7	21.5	23.9	45.6
C_16:0_	1.3	2.7	3.1	1.6
Anteiso-C_17:0_	14.5	3.6	5.1	1.8

Strains: 1, CHu50b-6-2^T^; 2, *G. tibetensis* KCTC 29148^T^; 3, *F. faeni* KACC 20509^T^; 4, *L. cavernae* DSM 27960^T^. All data were from present study. Cells of all strains were harvested after growth on R2A agar at 30 °C for 48 h. tr, not detected or less than 1%

**Table 3:** Differential characteristics of strain CHu50b-6-2^T^ and members of related genera in the family *Microbacteriaceae*

Characteristics	1	2	3	4	5	6	7	8	9	10
Pigmentation	Yellow	Yellow	Yellow	Yellow	Cream	Coral, yellow, pink	Yellow	White	Yellow	White, yellow
Motility	–	–	v	–	–	v	+	–	–	v
Diamino acid(s)	DAB	DAB	d-Lys	l-Lys	Lys	DAB	d-Orn	l-DAB	Lys, Orn	Orn
Peptidoglycan	B1*α*	B10	B2*β*	unknow peptidoglycan structure	B2*β*	B2*γ*	B2*β*	B1	B	B2*β*
Respiratory quinones	MK-10	MK-9, 10, 11	MK-9	MK-9, 10, 11	MK-9	MK-8, 9, 10, 11, 12	MK-9, 10, 11, 12, 13	MK-11, 12	MK-10, 11	MK-7, 8, 9
Major polar lipids	DPG, PG, GL	DPG, PG, GL	DPG, PG, GL	DPG, PG, GL, PL, L	DPG, PG, GL	DPG, PG, L	DPG, PG, GL	DPG, PG, GL	PG, DPG	PG, GL
DNA G + C content (mol%)	67.3	64.1–66.9	68.2–71.0	62.6	68	64.7–70	66	68.0	61.0–63.5	65.4–71

Taxa: 1, Strain CHu50b-6-2^T^ (from this study); 2, *Glaciihabitans* (Li et al. 2014; Dahal and Kim 2019); 3, *Frigoribacterium* (Kämpfer et al. 2000; Wang et al. 2015; Kong et al. 2016); 4, *Lysinibacter* (Tuo et al. 2015); 5, *Parafrigoribacterium* (Dastager et al. 2008a; Kong et al. 2016); 6, *Cryobacterium* (Suzuki et al. 1997; Dastager et al. 2008b; Liu et al. 2013, 2018); 7, *Homoserinimonas* (Kim et al. 2012a); 8, *Compostimonas* (Kim et al. 2012b); 9, *Salinibacterium* (Han et al. 2003; Zhang et al. 2008); 10, *Frondihabitans* (Zhang et al. 2007; Greene et al. 2009; Lee 2010; Cardinale et al. 2011; Kim et al. 2014). −, negative; +, positive; v, variable

DAB, 4-diaminobutyric acid; Lys, lysine; d-Orn, d-ornithine; DPG, diphosphatidylglycerol; PG, phosphatidylglycerol; GL, unidentified glycolipid; L, unidentified lipid

On the basis of the phylogenetic position and genotypic, chemotaxonomic, and physiological differences, we propose that strain CHu50b-6-2^T^ should be assigned as a novel species within a new genus, *Lacisediminihabitans* gen. nov., with the name *Lacisediminihabitans profunda* sp. nov. within the family *Microbacteriaceae*.

The Digital Protologue database (Rosselló-Móra et al. 2017) TaxoNumber for type strain CHu50b-6-2^T^ is GA00113.

### Description of *Lacisediminihabitans profunda* gen. nov.

*Lacisediminihabitans* (La.ci.se.di.mi.ni.ha.bi*’*tans. L. n. *lacus* lake; L. n. *sedimeninis* sediment; L. masc. n. *habitans* an inhabitant; N.L. fem. n. *Lacisediminihabitans* an inhabitant of lake sediment).

Cells are observed to be Gram-stain-positive, non-spore-forming, non-motile and rod-shaped. The predominant respiratory menaquinone is MK-10. The major polar lipids are diphosphatidylglycerol (DPG), phosphatidylglycerol (PG), and an unidentified glycolipid (GL). The major fatty acids are *anteiso*-C_15:0_, *iso*-C_16:0_, and *anteiso*-C_17:0_. The cell-wall peptidoglycan is B1*α* contains 2, 4-diaminobutyric acid as the diagnostic diamino acid. Phylogenetically, the genus belongs to the family *Microbacteriaceae* in the class *Actinobacteria*, being closely related to the genera *Glaciihabitans*, *Frigoribacterium*, and *Lysinibacter*. The type species is *Lacisediminihabitans profunda*.

### Description of *Lacisediminihabitans profunda* sp. nov.

*Lacisediminihabitans profunda* (pro.fun’da. L. fem. adj. *profunda* from the deep).

In addition to the characteristics described above, the novel species has the following properties. Colonies on R2A are convex, circular with entire edges and yellow color. The cells are observed to be oxidase-negative but catalase-positive. Growth occurs on R2A at temperatures from 4 to 30 °C (optimum temperature 25–30 °C), but not at 37 °C. The pH range for growth is from pH 6–10 (optimum pH 7); however, there is no growth at pH 5 and 11. No growth was observed on TSA, LB, and NA media. The cells are positive for nitrate reduction and *β*-galactosidase but negative for aesculin hydrolysis, indole production, glucose fermentation, urease, arginine dihydrolase or gelatin hydrolysis (API 20NE test strip). The G + C content of the genomic DNA is 67.3 mol%.

The type strain is CHu50b-6-2^T^ (= KCTC 49081^T^ = JCM 32673^T^) isolated from a 67-cm-long sediment core taken from the Daechung Reservoir, Republic of South Korea.

## Electronic supplementary material

Below is the link to the electronic supplementary material.

Supplementary material 1 (PDF 1232 kb)
