# Supplementary material for: Lacisediminihabitans profunda gen. nov., sp. nov., a member of the family Microbacteriaceae isolated from freshwater sediment
Source: Antonie Van Leeuwenhoek. 2019 Oct 18;113(3):365–75. doi: 10.1007/s10482-019-01347-8 (PMC7033078; doi:10.1007/s10482-019-01347-8)
Supplement: Supplementary file 1 — Supplementary material 1 (PDF 1232 kb) [file 10482_2019_1347_MOESM1_ESM.pdf]

ARTICLE TITLE

Lacisediminihabitans profunda gen. nov., sp. nov., a member of the family *Microbacteriaceae* isolated from freshwater sediment

Ye Zhuo[1‡], Chun-Zhi Jin[2, 3‡], Feng-Jie Jin[1], Taihua Li[1], Dong Hyo Kang[2, 3], Hee-Mock Oh[4], Hyung-Gwan Lee[4]* & Long Jin[1]*

[1]College of Biology and the Environment, Co-Innovation Centre for Sustainable Forestry in Southern China, Nanjing Forestry University, Nanjing210037, China

[2]Department of Bio-Molecular Science, KRIBB School of Bioscience, Korea University of Science and Technology (UST), 217 Gajeong-ro, Yuseong-gu, Daejeon, Republic of Korea

[3]Industrial Biomaterial Research Center, Korea Research Institute of Bioscience & Biotechnology (KRIBB), Daejeon 34141, Republic of Korea

[4]Cell Factory Research Centre, Korea Research Institute of Bioscience & Biotechnology (KRIBB), Daejeon 34141, Republic of Korea

Running title: *Lacisediminihabitans profunda* gen. nov. sp. nov.

Subject Category: *Actinobacteria*

‡These authors contributed equally to this work.

***Corresponding authors**

Long Jin
Tel: +86-25-8542-7210
Fax: +86-25-8542-7210
e-mail: isacckim@kaist.ac.kr

Hyung-Gwan Lee
Tel: +82-42-860-4318
Fax: +82-42-860-4594
e-mail: trustin@kribb.re.kr

The GenBank/EMBL/DDBJ accession numbers for sequences generated in this study are as follows: MF770244 (16S rRNA), MK910353 (*rec*A), and VRMG00000000 (whole genome sequence).

Table S1. Features of draft genome sequence of type strain CHu50b-6-2^T.

Attribute	Value
Assembled genome size (bp)	4,022,930
Contig number	175
G + C content (%)	67.3%
N50	413,391
Coding sequence (no.)	3,975
16S rRNA genes (no.)	4
23S rRNA genes (no.)	1
5S rRNA genes (no.)	2
tRNA genes (no.)	48

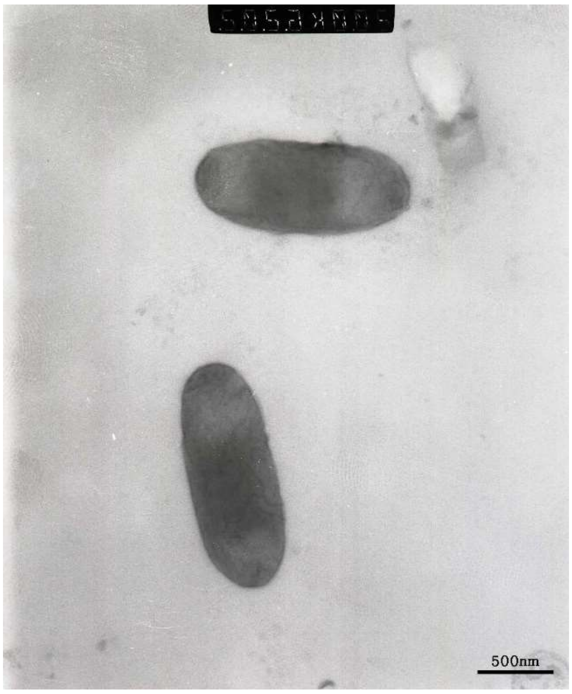

Supplementary Fig. S1. Transmission electron micrograph of strain CHu50b-6-2^{T} grown on R2A for 48 h at 30 °C. Bar, 0.5 μm.

(a)

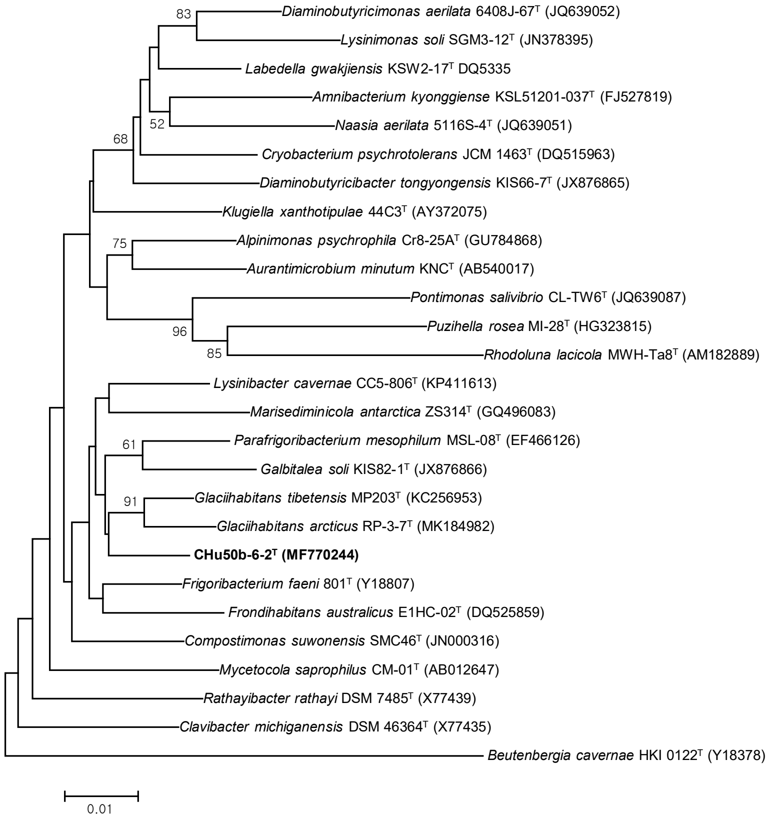

83
Diaminobutyricimonas aerilata 6408J-67T (JQ639052)
Lysinimonas soli SGM3-12T (JN378395)
Labedella gwakjiensis KSW2-17T DQ5335
Amnibacterium kyonggiense KSL51201-037T (FJ527819)
52
Naasia aerilata 5116S-4T (JQ639051)
68
Cryobacterium psychrotolerans JCM 1463T (DQ515963)
Diaminobutyricibacter tongyongensis KIS66-7T (JX876865)
Klugiella xanthotipulae 44C3T (AY372075)
75
Alpinimonas psychrophila Cr8-25AT (GU784868)
Aurantimicrobium minutum KNCT (AB540017)
Pontimonas salivibrio CL-TW6T (JQ639087)
96
Puzihella rosea MI-28T (HG323815)
85
Rhodoluna lacicola MWH-Ta8T (AM182889)
Lysinibacter cavernae CC5-806T (KP411613)
Marisediminicola antarctica ZS314T (GQ496083)
61
Parafrigoribacterium mesophilum MSL-08T (EF466126)
Galbitalea soli KIS82-1T (JX876866)
91
Glaciihabitans tibetensis MP203T (KC256953)
Glaciihabitans arcticus RP-3-7T (MK184982)
CHu50b-6-2T (MF770244)
Frigoribacterium faeni 801T (Y18807)
Frondihabitans australicus E1HC-02T (DQ525859)
Compostimonas suwonensis SMC46T (JN000316)
Mycetocola saprophilus CM-01T (AB012647)
Rathayibacter rathayi DSM 7485T (X77439)
Clavibacter michiganensis DSM 46364T (X77435)
Beutenbergia cavernae HKI 0122T (Y18378)
0.01

(b)

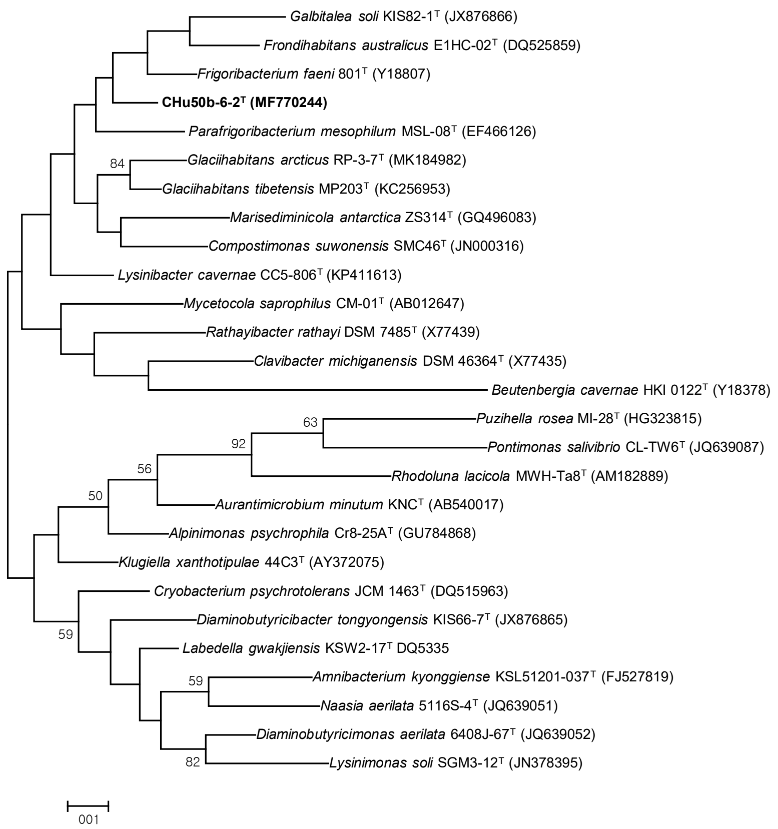

Supplementary Fig. S2. Neighbour-Joining (a) and Maximum-Parsimony (b) trees based on 16S rRNA gene sequences showing positions of strain CHu50b-6-2^{T} and related taxa. Only bootstrap values (expressed as percentages of 1000 replications) greater than 50 % are indicated at nodes. *Beutenbergia cavernae* HKI 0122^{T} (Y18378) was used as an outgroup. Bar, 1 substitutions per 100 nt.

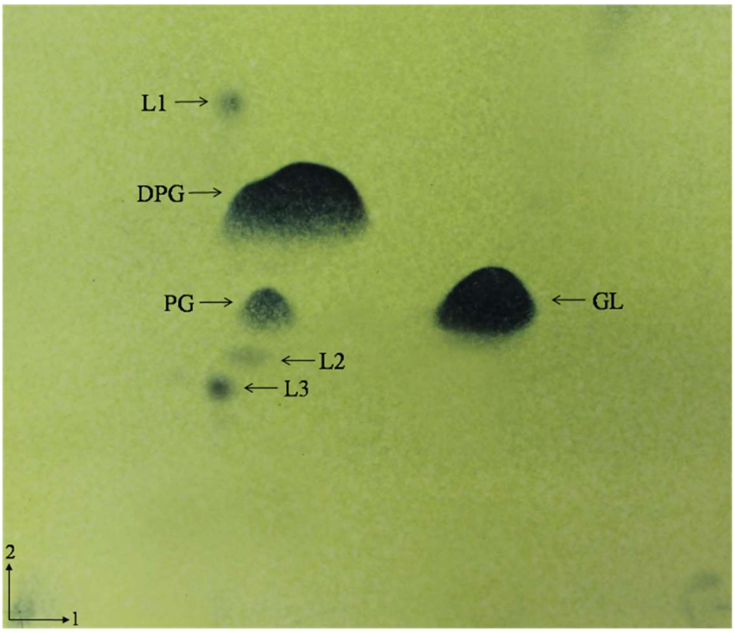

Supplementary Fig. S3. Polar lipid profile of strain CHu50b-6-2^T. All the polar lipids were stained with molybdophosphoric acid (for total lipids), molybdenum blue (for phospholipids), and ninhydrin (for amino lipids). Solvents were as follows: first direction, chloroform/methanol/water (65:25:4, by vol.); second direction, chloroform/methanol/acetic acid/water (40:7.5:6:1.8, by vol.). DPG, diphosphatidylglycerol; PG, phosphatidylglycerol; GL, unidentified glycolipid; L, unidentified lipids.
